# Laboratory Test Overuse and Compliance With Minimum Retesting Intervals in a Portuguese Healthcare Network

**DOI:** 10.7759/cureus.111275

**Published:** 2026-06-22

**Authors:** João A Pinto, Ana Dourado

**Affiliations:** 1 Clinical Pathology Department, Unidade Local de Saúde do Nordeste, Bragança, PRT

**Keywords:** clinical decision support, diagnostic stewardship, laboratory demand management, laboratory test overuse, minimum retesting intervals, test appropriateness

## Abstract

Background

Laboratory test overuse is a well-recognized challenge in modern healthcare, contributing to unnecessary costs, inefficient resource utilization, and potential patient harm. Inappropriate repeat testing, particularly outside recommended minimum retesting intervals (MRIs), represents a measurable target and actionable strategy to improve laboratory demand management. This study evaluated MRI compliance across multiple clinical settings and quantified the potential impact of MRI-based hard-stop restrictions.

Methodology

A retrospective observational study was conducted at a regional healthcare network in Northeast Portugal, including three hospitals and 14 primary care centers. All laboratory requests for 77 biomarkers performed between January 2023 and December 2024 were analyzed. Repeat tests were classified as appropriate or inappropriate based on established MRI recommendations. Patterns were compared across primary care, hospital outpatient, and inpatient/emergency settings. Statistical comparisons were performed using chi-square and z-tests for proportions. Potential cost savings were estimated based on reagent costs.

Results

Among 3,012,396 tests, 21.4% were performed outside recommended MRI thresholds. Inappropriateness rates were significantly different across care settings (p < 0.001): 6.7% in primary care, 15.4% in hospital outpatient care, and 31.4% in hospital inpatient care. Overutilization was particularly pronounced for frequently requested biomarkers, as 10 of the 11 most frequently prescribed biomarkers showed overall inappropriateness rates ranging from 24.4% to 32.5%. The simulated implementation of MRI-based hard-stop rules was projected to reduce laboratory expenditure by 14.2% (€119,281.93 annually). Notably, 38.5% of the estimated savings were attributable to B-type natriuretic peptide alone.

Conclusions

Substantial non-compliance with MRI recommendations was identified across all levels of care, particularly in the hospital inpatient setting. These findings highlight the need for targeted laboratory demand management strategies, especially in high-intensity clinical environments. MRI-based interventions, including electronic decision-support tools such as hard-stop policies, may represent effective and sustainable approaches to improve test appropriateness, reduce healthcare costs, and minimize patient harm associated with unnecessary diagnostic cascades.

## Introduction

Laboratory tests are essential in modern medicine, guiding diagnosis, monitoring, and therapeutic decision-making. However, test overuse is widespread and represents a growing challenge for healthcare systems, leading to unnecessary costs, inefficient resource utilization, downstream investigations, and potential patient harm, including overdiagnosis, iatrogenesis, and prolonged hospital stays. Evidence suggests up to 20% of tests may be redundant, particularly when repeated without a clear clinical indication or a consideration of biological and analytical variation [1-5].

Drivers of test overuse are multifactorial, including variability in professional training, diagnostic uncertainty, fear of litigation, limited awareness of test costs, and the absence of institutional standards for test ordering and repetition [2-4]. While inappropriate initial testing is context-dependent, unnecessary test repetition is objectively measurable and represents a suitable target for intervention. One of the most widely adopted strategies to address this issue is the implementation of minimum retesting intervals (MRIs), which have been defined for numerous biomarkers based on biological variability, clinical relevance, and expert consensus [6-20]. Operationalizing these recommendations in clinical practice, however, requires effective demand management strategies.

Several interventions have been proposed to support laboratory demand management, including audit and feedback, cost transparency, professional education, optimization of electronic ordering systems, and institutional policy changes [1-5]. Among these, electronic clinical decision-support strategies, including hard-stop and soft-stop restrictions, reflex testing algorithms, and specialized review of test requests, have shown the most consistent and sustainable reductions in inappropriate test ordering [1]. Hard-stop restrictions prevent the ordering of a test before a predefined time interval has elapsed. While other strategies may complement laboratory demand management, hard-stop restrictions provide a direct and objective mechanism to enforce and evaluate compliance with MRI. Importantly, available evidence indicates that such interventions do not increase adverse events or compromise clinical outcomes [3-5].

Despite the growing adoption of MRI-based strategies and the extensive literature defining MRI recommendations for different biomarkers and clinical contexts, studies evaluating real-world compliance with these recommendations remain relatively limited. Published studies are highly heterogeneous and have generally focused on a limited number of biomarkers, individual hospital departments, or a single clinical setting, most commonly inpatient populations [9-11,13,16-18]. Consequently, there is limited large-scale evidence describing how compliance with MRI recommendations varies across different levels of care within the same healthcare system or identifying which biomarkers contribute most substantially to inappropriate repeat testing and its associated economic burden.

In this context, the present study evaluates compliance with MRI recommendations across a broad panel of laboratory biomarkers within a regional healthcare network, using data from repeated laboratory test requests performed between 2023 and 2024. Specifically, the study characterizes laboratory testing practices across different levels of care, identifies the biomarkers contributing most substantially to inappropriate repeat testing, and estimates the potential financial impact of simulated MRI-based hard-stop restrictions.

## Materials and methods

This retrospective observational study aimed to assess compliance with MRI recommendations at Unidade Local de Saúde do Nordeste (ULSNE), a public healthcare institution in Northeast Portugal serving approximately 128,000 inhabitants. ULSNE comprises three hospitals with a total capacity of 431 beds (including 18 intensive care unit beds) and 14 primary healthcare centers.

Over a 24-month period, from January 2023 to December 2024, all laboratory requests for 77 selected biomarkers were analyzed to evaluate patterns of test repetition. Biomarkers were included based on the availability of MRI recommendations in the literature. When multiple recommendations were available, thresholds were selected based on concordance among published sources, clinical plausibility, and applicability to local laboratory practice. For serological tests without clearly defined MRI recommendations, a conservative 30-day interval was applied, based on the typical timeframe required for seroconversion (generally 2-4 weeks).

Data were extracted from the laboratory information system and anonymized before analysis. No patient medical records or clinical information were consulted. For each patient, consecutive requests for the same biomarker were identified throughout the study period, irrespective of clinical encounter or requesting department. Repeat requests were classified as appropriate or inappropriate based on the time elapsed between consecutive requests and the corresponding MRI recommendation. This approach aimed to capture patterns of potential analytical overuse representative of real-world clinical practice. Cost savings were estimated by multiplying the number of inappropriate tests by the unit reagent cost for each biomarker.

Three groups were defined according to the clinical setting: (1) primary healthcare, (2) hospital outpatient care (specialist consultations), and (3) hospital inpatient care (including admitted patients and emergency department visits). Emergency department requests were grouped with inpatient testing because both represent acute-care settings characterized by rapid clinical decision-making and greater diagnostic uncertainty. Outpatient requests were separated according to clinical context. Primary care is typically oriented toward preventive medicine and long-term monitoring, whereas hospital outpatient consultations are more frequently focused on diagnosis and treatment. Nevertheless, the same MRI thresholds were applied to both outpatient settings, in accordance with recommendations reported in the literature. Biomarkers were analyzed individually and grouped into broad physiological categories to explore potential patterns of test repetition across related analytes.

The primary outcome was compliance with MRI recommendations for each biomarker across the three clinical settings. Secondary objectives were to estimate the potential reduction in test volume and laboratory expenditure associated with the simulated implementation of MRI-based hard-stop rules.

At the time of the study, ULSNE had no analytical restriction rules, electronic decision-support alerts, or automated enforcement mechanisms related to MRI recommendations. No educational or training interventions were conducted by the authors targeting prescribing clinicians.

The study was conducted in accordance with institutional and national regulations for retrospective observational research. Given its retrospective design and the use of anonymized data, informed consent was not required.

Statistical analysis

Data were analysed using SPSS® Statistics (IBM Corp., Armonk, NY, USA). Descriptive statistics were used throughout, with results reported as absolute frequencies and percentages according to biomarker and clinical setting. The unit of analysis was individual laboratory test requests. Compliance with MRI recommendations was defined as the proportion of repeat test requests performed at or beyond the recommended interval for each biomarker. Differences in inappropriateness rates between clinical settings were assessed using chi-square tests of independence. Pairwise comparisons of proportions were performed using z-tests for independent samples. All tests were two-sided, with statistical significance set at p-values <0.05. No formal adjustment for multiple comparisons was applied given the exploratory nature of subgroup analyses.

## Results

Over the two-year study period, a total of 3,012,396 laboratory analyses were performed for the selected biomarkers (Table 1), providing a large dataset for evaluation of test repetition patterns across clinical settings. Of these, 54.6% (n = 1,643,482) were requested in the hospital inpatient setting, 13.9% (n = 420,101) in the hospital outpatient setting, and 31.5% (n = 948,813) in the primary healthcare setting.

**Table 1:** MRI non-compliant repeat laboratory requests and projected annual cost savings under a simulated hard-stop MRI strategy. MRI = minimum retesting interval; beta-hCG = beta-human chorionic gonadotropin; HDL = high-density lipoprotein; ALT = alanine aminotransferase; AST = aspartate aminotransferase; GGT = gamma-glutamyl transferase; CA 125 = carbohydrate antigen 125; CA 15-3 = carbohydrate antigen 15-3; CA 19-9 = carbohydrate antigen 19-9; CEA = carcinoembryonic antigen; PSA = prostate-specific antigen; CCP = cyclic citrullinated peptide; CMV = cytomegalovirus; EBV EBNA = Epstein-Barr virus nuclear antigen; EBV VCA = Epstein–Barr virus viral-capsid antigen; HAV = hepatitis A virus; HBc = hepatitis B core antigen; HBe = hepatitis B e-antibody; HBs = hepatitis B surface antibody; HCV = hepatitis C virus

Categories	Healthcare network		Biomarkers	Inpatients MRI	Outpatients/Primary care MRI	Primary care		Hospital outpatients		Hospital inpatients		Healthcare network		
	Non-compliant repetitions	Total				Non-compliant repetitions	Total	Non-compliant repetitions	Total	Non-compliant repetitions	Total	Non-compliant repetitions	Total	Annualized savings
Cardiac biomarkers	9,228 (32.8%)	28,157	B-Type Natriuretic Peptide	7 days	180 days	73 (15.9%)	460	870 (37.7%)	2,306	8,285 (32.6%)	25,391	9,228 (32.8%)	28,157	€45,955.44
Electrolytes	84,336 (28.5%)	295,810	Bicarbonate	4 days	30 days	0 (0.0%)	18	23 (1.0%)	2,197	31 (4.1%)	747	54 (1.8%)	2,962	€5.94
			Total Calcium	4 days	30 days	104 (1.6%)	6,376	587 (8.6%)	6,794	5,558 (35.1%)	15,848	6,249 (21.5%)	29,018	€281.21
			Phosphorus	4 days	30 days	48 (2.7%)	1,804	507 (8.9%)	5,712	8,154 (46.5%)	17,539	8,709 (34.8%)	25,055	€479.00
			Ionogram (Na, K, Cl)	4 days	30 days	2,856 (6.4%)	44,458	3,729 (12.7%)	29,288	53,670 (38.6%)	139,174	60,255 (28.3%)	212,920	€1,205.10
			Magnesium	4 days	30 days	83 (2.8%)	2,987	508 (12.3%)	4,128	8,478 (45.2%)	18,740	9,069 (35.1%)	25,855	€634.83
Endocrine biomarker	7,419 (11.8%)	63,024	Beta-hCG	7 days		10 (3.5%)	288	5 (3.0%)	166	48 (7.4%)	649	63 (5.7%)	1,103	€58.28
			HbA1c	90 days		3,278 (8.4%)	38,921	1,652 (18.7%)	8,839	1,727 (26.6%)	6,483	6,657 (12.3%)	54,243	€4,659.90
			Parathyroid hormone	90 days		116 (4.0%)	2,872	281 (7.8%)	3,607	302 (25.2%)	1,199	699 (9.1%)	7,678	€772.40
Enzymatic biomarker	27,279 (30.4%)	89,626	Lactate dehydrogenase	3 days	90 days		5,032	1,988 (26.2%)	7,602	24,788 (32.2%)	76,992	27,279 (30.4%)	89,626	€1,091.16
Hematological biomarkers	65,847 (26.1%)	252,514	Complete blood count	4 days	30 days	5,528 (7.4%)	75,038	4,887 (14.4%)	33,828	55,432 (38.6%)	143,648	65,847 (26.1%)	252,514	€18,766.40
Iron biomarkers	16,808 (17.4%)	96,520	Ferritin	90 days		1,139 (7.8%)	14,643	860 (13.7%)	6,287	3,391 (39.8%)	8,519	5,390 (18.3%)	29,449	€4,985.75
			Iron	90 days		1,438 (7.6%)	18,940	1,422 (19.1%)	7,462	3,221 (37.0%)	8,700	6,081 (17.3%)	35,102	€334.46
			Transferrin	90 days		317 (5.7%)	5,605	166 (10.5%)	1,584	1,029 (27.6%)	3,727	1,512 (13.9%)	10,916	€816.48
			Unsaturated iron binding capacity	90 days		563 (7.0%)	8,036	826 (14.8%)	5,576	2,436 (32.7%)	7,441	3,825 (18.2%)	21,053	€420.75
Lipids	22,796 (8.6%)	266,478	HDL cholesterol	90 days		3,916 (5.6%)	69,382	1,657 (14.6%)	11,329	1,288 (21.7%)	5,934	6,861 (7.9%)	86,645	€1,063.46
			Total cholesterol	90 days		4,241 (6.1%)	69,991	2,366 (16.6%)	14,223	1,400 (22.6%)	6,206	8,007 (8.9%)	90,420	€280.25
			Triglycerides	90 days		4,123 (6.0%)	69,000	2,342 (16.6%)	14,138	1,463 (23.3%)	6,275	7,928 (8.9%)	89,413	€317.12
Liver biomarkers	257,387 (28.6%)	899,754	Albumin	3 days	90 days	176 (7.8%)	2,254	1,570 (16.6%)	9,447	9,599 (34.2%)	28,032	11,345 (28.6%)	39,733	€794.15
			ALT	3 days	90 days	6,947 (12.6%)	55,253	5,410 (29.1%)	18,605	33,271 (29.3%)	113,373	45,628 (24.4%)	187,231	€1,596.98
			AST	3 days	90 days	7,121 (12.6%)	56,542	5,409 (29.1%)	18,588	33,456 (29.4%)	113,630	45,986 (24.4%)	188,760	€1,609.51
			Direct bilirubin	7 days	90 days	1,018 (10.8%)	9,425	2,585 (25.7%)	10,055	31,820 (41.4%)	76,770	35,423 (36.8%)	96,250	€2,125.38
			Total bilirubin	3 days	90 days	1,197 (10.5%)	11,355	2,644 (26.1%)	10,146	23,553 (30.1%)	78,290	27,394 (27.5%)	99,791	€958.79
			Alkaline phosphatase	7 days	90 days	2,198 (10.3%)	21,267	3,084 (26.5%)	11,642	31,316 (39.3%)	79,727	36,598 (32.5%)	112,636	€2,012.89
			GGT	7 days	90 days	4,469 (10.6%)	42,102	3,917 (24.9%)	15,700	34,554 (40.9%)	84,565	42,940 (30.2%)	142,367	€1,932.30
			Total proteins	7 days	90 days	144 (5.3%)	2,723	1,187 (14.5%)	8,163	10,742 (48.6%)	22,100	12,073 (36.6%)	32,986	€422.56
Neoplastic biomarkers	6,788 (13.2%)	51,235	CA 125	30 days		5 (0.4%)	1,407	22 (3.2%)	691	174 (20.9%)	832	201 (6.9%)	2,930	€185.93
			CA 15-3	30 days		12 (7.2%)	166	105 (10.5%)	998	396 (29.6%)	1,338	513 (20.5%)	2,502	€474.53
			CA 19-9	30 days		14 (1.5%)	965	274 (15.7%)	1,742	1,703 (51.7%)	3,297	1,991 (33.2%)	6,004	€1,841.68
			CEA	30 days		27 (2.3%)	1,152	376 (16.6%)	2,264	2,030 (50.8%)	4,000	2,433 (32.8%)	7,416	€2,250.53
			Free PSA	45 days		95 (2.0%)	4,760	74 (3.5%)	2,121	62 (8.4%)	737	231 (3.0%)	7,618	€184.80
			Total PSA	45 days		588 (3.4%)	17,332	490 (8.8%)	5,593	341 (18.5%)	1,840	1,419 (5.7%)	24,765	€1,135.20
Pancreatic biomarkers	5,146 (9.6%)	53,522	Amylase	1 day	7 days	21 (0.8%)	2,739	5 (0.8%)	604	378 (1.4%)	26,936	404 (1.3%)	30,279	€68.68
			Lipase	7 days	180 days	81 (19.6%)	414	114 (29.7%)	384	4,547 (20.3%)	22,445	4,742 (20.4%)	23,243	€1,114.37
Proteins	5,763 (2.9%)	201,231	Alpha-Fetoprotein	90 days		8 (2.0%)	402	21 (2.3%)	903	119 (9.2%)	1,293	148 (5.7%)	2,598	€127.28
			C3	90 days		3 (3.2%)	93	156 (7.4%)	2,103	155 (16.5%)	939	314 (10.0%)	3,135	€135.02
			C4	90 days		3 (3.2%)	93	157 (7.5%)	2,100	155 (16.5%)	939	315 (10.1%)	3,132	€149.63
			Protein electrophoresis	90 days		31 (2.4%)	1,294	199 (6.7%)	2,986	288 (15.4%)	1,873	518 (8.4%)	6,153	€1,004.92
			Rheumatoid factor	180 days		51 (4.2%)	1,208	349 (25.6%)	1,365	36 (8.7%)	412	436 (14.6%)	2,985	€176.58
			Immunoglobulin A	90 days		10 (2.6%)	388	181 (6.6%)	2,734	320 (16.6%)	1,931	511 (10.1%)	5,053	€242.73
			Total immunoglobulin E	90 days		4 (0.6%)	694	14 (1.9%)	736	12 (3.0%)	403	30 (1.6%)	1,833	€236.85
			Immunoglobulin G	90 days		10 (3.5%)	283	180 (6.7%)	2,687	332 (17.2%)	1,935	522 (10.6%)	4,905	€224.46
			Immunoglobulin M	90 days		6 (2.2%)	269	177 (6.7%)	2,626	311 (16.6%)	1,877	494 (10.4%)	4,772	€234.65
			Procalcitonin	1 day		0 (0.0%)	6	0 (0.0%)	828	248 (0.9%)	26,346	248 (0.9%)	27,180	€1,174.28
			C-reactive protein	1 day	7 days	108 (1.4%)	7,779	176 (2.1%)	8,214	1,943 (1.6%)	123,492	2,227 (1.6%)	139,485	€456.54
Renal biomarkers	120,437 (27.1%)	443,638	Creatinine	4 days	30 days	4,512 (6.6%)	68,362	4,368 (13.5%)	32,323	53,160 (38.0%)	139,803	62,040 (25.8%)	240,488	€1,551.00
			Urea	4 days	30 days	2,066 (6.1%)	33,725	3,596 (11.8%)	30,355	52,735 (37.9%)	139,070	58,397 (28.7%)	203,150	€1,459.93
Serological biomarkers	3,840 (5.2%)	73,187	Anti-CCP antibodies	180 days		14 (2.8%)	498	335 (28.7%)	1,168	7 (3.7%)	188	356 (19.2%)	1,854	€590.96
			Anti-CMV IgG antibodies	30 days		11 (1.1%)	1,013	2 (1.0%)	196	20 (3.9%)	518	33 (1.9%)	1,727	€30.53
			Anti-CMV IgM antibodies	30 days		11 (1.1%)	1,013	2 (1.0%)	196	20 (3.9%)	519	33 (1.9%)	1,728	€30.53
			Anti-EBV EBNA IgG antibodies	30 days		0 (0.0%)	49	1 (1.3%)	78	20 (5.4%)	372	21 (4.2%)	499	€30.24
			Anti-EBV VCA IgG antibodies	30 days		0 (0.0%)	49	1 (1.3%)	77	20 (5.4%)	372	21 (4.2%)	498	€30.24
			Anti-EBV VCA IgM antibodies	30 days		0 (0.0%)	49	1 (1.3%)	77	20 (5.4%)	373	21 (4.2%)	499	€30.24
			Anti-HAV IgG antibodies	30 days		0 (0.0%)	490	0 (0.0%)	81	1 (1.8%)	55	1 (0.2%)	626	€1.23
			Anti-HAV IgM antibodies	30 days		0 (0.0%)	492	0 (0.0%)	72	5 (1.6%)	309	5 (0.6%)	873	€4.63
			Anti-HBc total antibodies	30 days		1 (0.1%)	831	4 (0.3%)	1,351	53 (2.6%)	2,037	58 (1.4%)	4,219	€46.40
			Anti-HBe antibodies	30 days		0 (0.0%)	369	0 (0.0%)	40	2 (0.6%)	322	2 (0.3%)	731	€1.85
			Anti-HBs antibodies	30 days		4 (0.3%)	1,545	15 (0.6%)	2,368	64 (2.9%)	2,234	83 (1.4%)	6,147	€71.38
			Anti-HCV antibodies	30 days		14 (0.4%)	3,227	11 (0.6%)	1,943	71 (2.9%)	2,416	96 (1.3%)	7,586	€88.80
			Anti-HIV antibodies	30 days		50 (0.8%)	6,124	27 (1.4%)	1,909	85 (3.5%)	2,409	162 (1.6%)	10,442	€149.85
			Anti-microsomal antibodies	365 days		548 (15.1%)	3,636	623 (49.5%)	1,258	38 (14.8%)	256	1,209 (23.5%)	5,150	€967.20
			Anti-rubella IgG antibodies	30 days		20 (1.3%)	1,568	3 (2.7%)	113	2 (1.3%)	152	25 (1.4%)	1,833	€21.50
			Anti-rubella IgM antibodies	30 days		21 (1.3%)	1,568	2 (1.8%)	113	2 (1.3%)	152	25 (1.4%)	1,833	€21.50
			Anti-thyroglobulin antibodies	365 days		547 (15.0%)	3,635	633 (49.9%)	1,268	42 (16.2%)	260	1,222 (23.7%)	5,163	€977.60
			Anti-Toxoplasma IgG antibodies	30 days		46 (1.8%)	2,532	11 (4.6%)	239	9 (3.2%)	280	66 (2.2%)	3,051	€58.74
			Anti-Toxoplasma IgM antibodies	30 days		46 (1.8%)	2,531	11 (4.6%)	239	9 (3.2%)	280	66 (2.2%)	3,050	€58.74
			HBe antigen	30 days		0 (0.0%)	310	0 (0.0%)	54	3 (1.7%)	173	3 (0.6%)	537	€2.78
			HBs antigen	30 days		31 (0.7%)	4,360	23 (1.1%)	2,042	147 (5.5%)	2,692	201 (2.2%)	9,094	€172.86
			Treponemal test	30 days		40 (0.9%)	4,239	20 (2.5%)	808	71 (7.1%)	1,000	131 (2.2%)	6,047	€80.57
Thyroid biomarkers	5,196 (4.1%)	125,517	Free T3	30 days		18 (2.0%)	909	30 (2.5%)	1,180	71 (5.9%)	1,200	119 (3.6%)	3,289	€73.19
			Total T3	30 days		42 (0.7%)	6,108	22 (1.3%)	1,674	35 (3.9%)	887	99 (1.1%)	8,669	€60.89
			Free T4	30 days		490 (1.5%)	33,681	245 (3.7%)	6,698	1,302 (16.5%)	7,892	2,037 (4.2%)	48,271	€1,252.76
			Total T4	30 days		4 (2.2%)	186	13 (1.2%)	1,053	38 (3.9%)	977	55 (2.5%)	2,216	€33.83
			Thyroid-stimulating hormone	30 days		770 (1.7%)	45,765	373 (4.6%)	8,104	1,743 (18.9%)	9,203	2,886 (4.6%)	63,072	€1,601.73
Vitamins	5,157 (7.1%)	72,183	Folic acid	60 days		554 (3.1%)	18,047	406 (6.6%)	6,195	1,416 (20.9%)	6,777	2,376 (7.7%)	31,019	€1,829.52
			Vitamin B12	60 days		596 (3.2%)	18,687	399 (6.5%)	6,095	1,492 (21.1%)	7,069	2,487 (7.8%)	31,851	€2,300.48
			Vitamin D	90 days		143 (2.0%)	6,999	91 (5.5%)	1,643	60 (8.9%)	671	294 (3.2%)	9,313	€651.21
Total						63,282 (6.7%)	948,813	64,820 (15.4%)	420,101	515,325 (31.4%)	1,643,482	643,427 (21.4%)	3,012,396	€119,281.93 (14.2%)

The overall rate of analytical prescription inappropriateness at ULSNE was 21.4% (n = 643,427), with substantial variation across biomarkers, ranging from 0.2% to 36.8%. Inappropriateness rates differed significantly between the three clinical settings (χ² test, p < 0.001). In the primary healthcare setting, the overall inappropriateness rate was 6.7% (n = 63,282) (range across biomarkers: 0-19.6%), and only 10 of 77 biomarkers (13.0%) showed an inappropriateness rate exceeding 10%. In the hospital outpatient setting, the overall rate was 15.4% (n = 64,820) (range: 0-49.9%), with 31 of 77 biomarkers (40.3%) exceeding the 10% threshold. The highest level of non-compliance was observed in the hospital inpatient setting, where the inappropriateness rate reached 31.4% (n = 515,325) (range: 0.6-51.7%), and 44 of 77 biomarkers (57.1%) had rates above 10%. Pairwise comparisons confirmed that the three groups differed significantly from each other (z-test for proportions, p < 0.001). This pattern was evident for most biomarkers, including iron studies (e.g., ferritin: 7.8% vs. 13.7% vs. 39.8%), thyroid function tests (e.g., thyroid-stimulating hormone: 1.7% vs. 4.6% vs. 18.9%), and HbA1c (8.4% vs. 18.7% vs. 26.6%). Marked overutilization was identified for several biomarkers in the inpatient setting, particularly neoplastic biomarkers such as carcinoembryonic antigen (50.8%; n = 2,030) and carbohydrate antigen 19-9 (51.7%; n = 1,703). In contrast, some biomarkers showed distinct patterns, with higher inappropriateness rates in hospital outpatient settings, such as rheumatoid factor (25.6%; n = 349) and anti-cyclic citrullinated peptide antibodies (28.7%; n = 335), suggesting specialty-driven testing behaviors.

**Figure 1:**
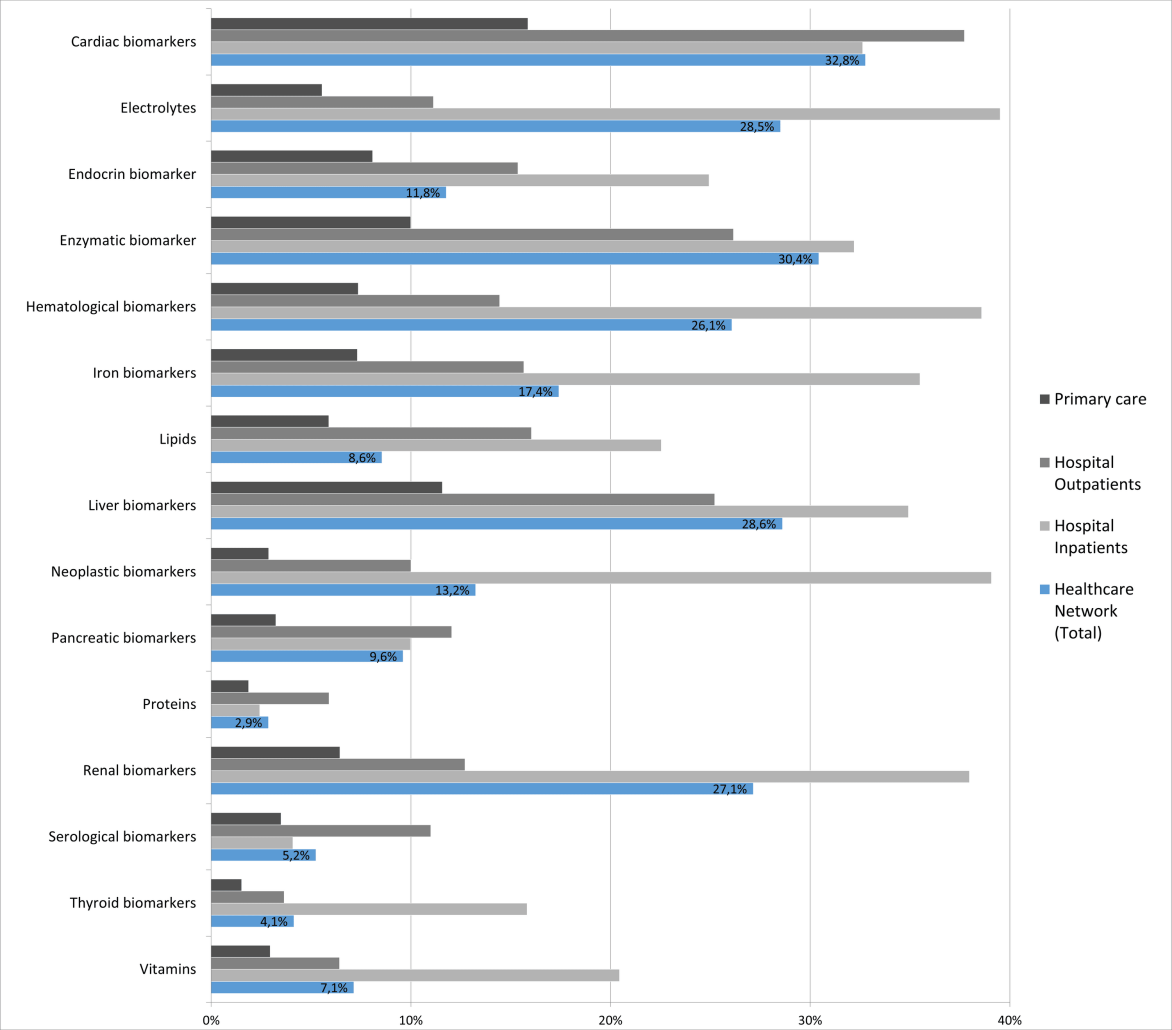
Inappropriateness rates according to biomarker category and clinical setting based on minimum retesting interval recommendations.

When biomarkers were grouped according to physiological categories (Figure 1), the lowest inappropriateness rates were observed for protein biomarkers (2.9%; n = 5,763), followed by thyroid biomarkers (4.1%; n = 5,196) and serological biomarkers (5.2%; n = 3,840). Intermediate levels of inappropriateness were identified for vitamins, lipids, pancreatic biomarkers, endocrine biomarkers, and neoplastic biomarkers, with overall rates ranging from 7.1% to 13.2%, while iron biomarkers showed an inappropriateness rate of 17.4% (n = 16,808). In contrast, the remaining categories, including hematological biomarkers, renal biomarkers, electrolytes, liver biomarkers, enzymatic biomarkers, and cardiac biomarkers, presented substantially higher rates, ranging from 26.1% to 32.8%. These findings suggest that test repetition patterns vary substantially according to the physiological class of biomarkers.

The most frequently requested biomarkers also tended to show relatively high inappropriateness rates. Specifically, 10 of the 11 most frequently prescribed biomarkers, i.e., complete blood count, creatinine, ionogram, urea, aspartate aminotransferase, alanine aminotransferase, gamma-glutamyltransferase, alkaline phosphatase, total bilirubin, and direct bilirubin, showed overall inappropriateness rates ranging from 24.4% to 32.5%. The exception was C-reactive protein, which, although it was the eighth most requested test, only had an inappropriateness rate of 1.6% (n = 2,227), despite a relatively short MRI threshold (one day for inpatients and seven days for outpatients).

**Figure 2:**
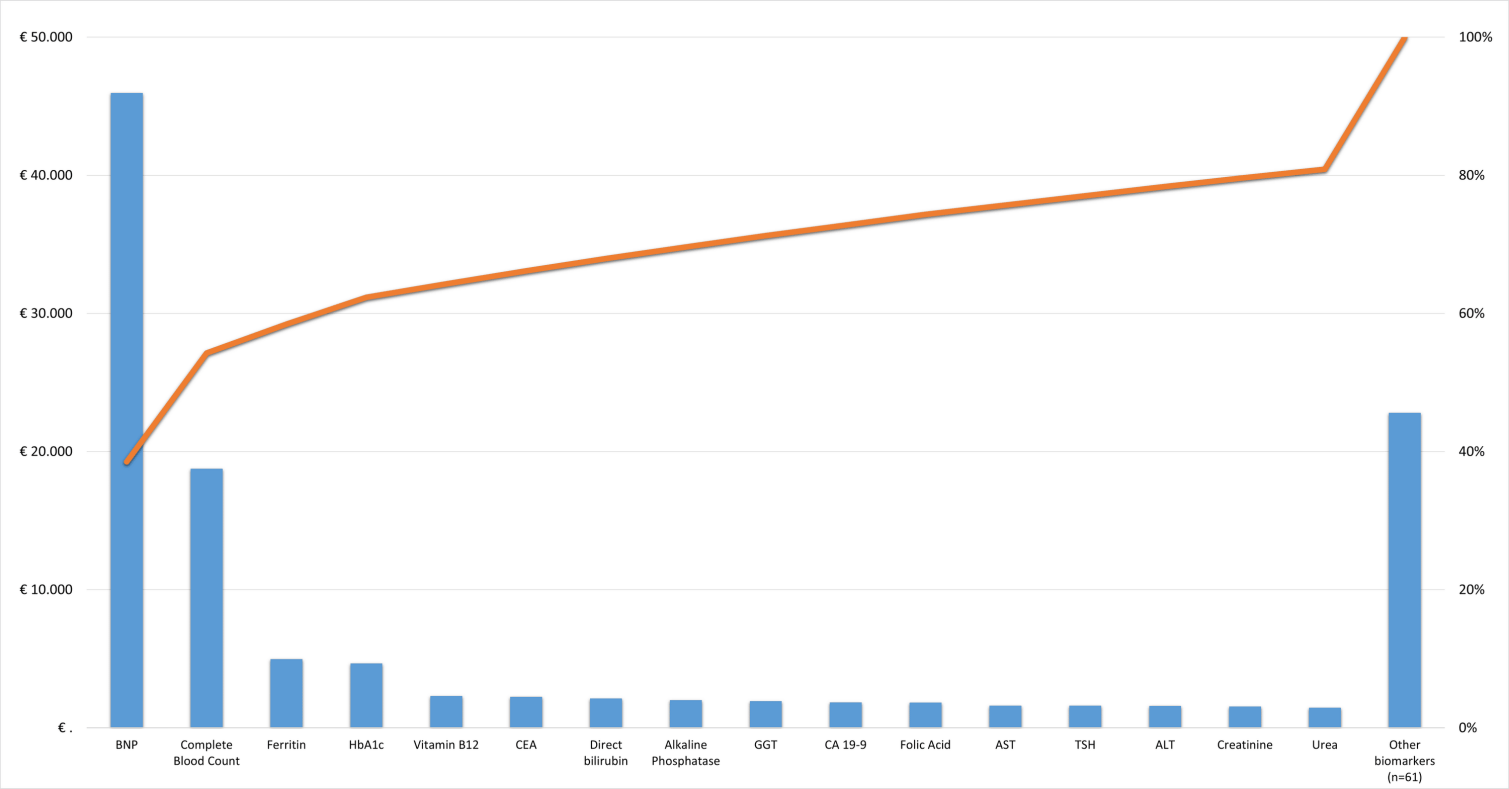
Cumulative contribution of individual biomarkers to projected annual cost savings under a simulated minimum retesting interval-based hard-stop strategy.

From an economic perspective, the simulated implementation of MRI-based hard-stop rules would result in an estimated 14.2% reduction in total laboratory expenditure (€119,281.93 per year). Notably, 38.5% of the projected cost savings would be attributable to improved prescribing of B-type natriuretic peptide (BNP) alone, followed by complete blood count (15.7%), ferritin (4.2%), and HbA1c (3.9%). A Pareto analysis showed that 80.9% of the projected annual cost savings were attributable to only 16 biomarkers, whereas the remaining 61 biomarkers accounted for just 19.1% of the estimated savings (Figure 2).

## Discussion

MRIs are grounded in established principles of biological variation and clinical utility. Repeating a laboratory test before a meaningful physiological or clinical change is unlikely to influence clinical management and may contribute to downstream consequences, including additional investigations, incidental findings, and prolonged hospitalization. Although MRI recommendations cannot account for every individual scenario and cannot replace clinical judgment, they are designed to be broadly applicable to most situations encountered in routine clinical practice and therefore represent a valid strategy for laboratory demand management.

MRI thresholds were selected based on published literature, prioritizing more restrictive criteria to better capture clinically meaningful overuse, as permissive criteria tend to underestimate overall inappropriateness [2]. This methodological choice, while representing a limitation, aligns with the objective of identifying systemic drivers of unnecessary test repetition rather than subjectively evaluating individual prescribing decisions.

This study demonstrates substantial non-compliance with published MRI recommendations across different levels of care within our healthcare network, with an overall inappropriateness rate of 21.4%, which is consistent with the literature [1-5]. A clear gradient was observed, ranging from 6.7% in primary healthcare to 15.4% in hospital outpatients and reaching 31.4% in the inpatient setting. More than half of the biomarkers in the hospital inpatient setting exceeded a 10% non-compliance threshold, compared with only 13.0% in primary care. This progressive increase from primary care to inpatient care likely reflects differences in clinical complexity, diagnostic uncertainty, workflow organization, and institutional culture. In hospital environments, particularly inpatient care, test repetition may be driven by routine daily ordering practices, defensive medicine, and insufficient review of previous results. In contrast, primary care generally involves more structured follow-up intervals and less frequent immediate reassessment, which may partly explain the lower non-compliance rates observed.

The hospital outpatient setting presented overall intermediate levels of inappropriateness compared with the other two clinical contexts. Despite the heterogeneous clinical environments, the same MRI thresholds were applied as in primary care, as both involve outpatient management. Current MRI recommendations do not distinguish between these two settings; however, they were analyzed separately in this study because they represent distinct clinical environments. Notably, statistically significant differences were identified for several biomarkers. These differences may partly reflect variations in clinical context, but they may also be influenced by other factors, such as the replication of laboratory ordering patterns by physicians across hospital environments, regardless of whether the context is inpatient or outpatient. This interpretation is supported by findings showing that for some biomarkers there are significant differences between clinical settings (e.g., HbA1c), even though the MRI thresholds are the same. In addition, repetition may also be influenced by potential limitations in system interoperability, although this could not be directly assessed in the present study.

Although laboratory demand management and MRI implementation have been explored previously, direct comparison with published studies remains challenging because of substantial methodological heterogeneity. Published studies differ considerably with respect to the biomarkers evaluated, MRI thresholds applied, clinical settings included, and the presence of concurrent demand-management interventions. Few published studies have included both inpatient and outpatient settings, with Boerman et al. representing one notable example [9]. This study evaluated MRI compliance in a Dutch hospital following the implementation of MRI-based restrictions within a broader stewardship program that also included educational and feedback interventions. Overall, only a small proportion of requests (1.59%) generated MRI alerts and ultimately resulted in test cancellation, a finding that may partly reflect the impact of the pre-existing educational and feedback interventions, which may have limited the ability to assess the isolated contribution of MRI-based restrictions. Furthermore, the analysis was restricted to a limited number of biomarkers (28) and only included requests from physicians belonging to the internal medicine department. Similar methodological differences are present across other published studies, many of which have focused on individual departments, selected biomarkers, or specific patient populations [10-11,13,16-18].

To our knowledge, no previous study has evaluated compliance across such a broad panel of biomarkers (77 in total) while simultaneously comparing primary care, hospital outpatient, and inpatient settings within the same healthcare network. Importantly, no MRI-based restrictions, decision-support tools, or educational interventions were in place during the study period, suggesting that the observed patterns likely reflect baseline prescribing behavior under routine clinical practice conditions. By quantifying MRI non-compliance across multiple levels of care, our findings provide system-wide and locally relevant evidence regarding the burden of unnecessary laboratory test repetition and identify potential targets for future laboratory demand-management interventions.

Importantly, previous evidence suggests that electronic decision-support interventions, including hard-stop strategies, do not increase adverse events or compromise clinical outcomes when appropriately designed and monitored. Our findings therefore support the feasibility and potential value of incorporating systematic MRI-based restrictions into electronic ordering systems as part of broader laboratory demand management initiatives. Improved demand management may also contribute to shorter turnaround times and more efficient allocation of laboratory resources.

B-type natriuretic peptide

The clinical utility of natriuretic peptides in the diagnosis and prognostic stratification of heart failure is well established. However, multiple randomized controlled trials have failed to demonstrate superiority of BNP- or NT-proBNP-guided therapy over careful clinical assessment for routine monitoring of heart failure in both inpatient and outpatient settings [21-23]. Current evidence therefore supports titration of guideline-directed medical therapy primarily based on clinical status rather than serial biomarker measurements [24-27].

In hospitalized patients with heart failure, the available literature generally recommends limiting natriuretic peptide measurement to once per admission, except in specific contexts such as pre-discharge assessment. In the outpatient setting, the suggested MRI threshold is six months [6-8]. In our study, a seven-day MRI threshold was applied for inpatients to ensure an objective and reproducible cutoff. As most patients hospitalized for acute heart failure are unlikely to be discharged within one week, this threshold likely captures meaningful overprescription. The alternative would require detailed case-by-case evaluation to determine whether testing occurred in the context of pre-discharge assessment, introducing substantial subjectivity.

A particularly relevant finding of this study is that 38.5% of the projected cost savings from hypothetical hard-stop rules would be attributable to BNP alone. Notably, BNP was among the biomarkers with the lowest compliance with MRI recommendations across all clinical settings. The overall inappropriateness rate was 32.8%: 15.9% in primary care, 37.7% in hospital outpatients, and 32.6% in hospital inpatients. Among inpatients, BNP inappropriate prescribing differed significantly between clinical contexts, reaching 72.0% in intensive care units (based on the seven-day MRI threshold) compared to 25.6% in internal medicine and emergency department settings (z-test for proportions, p < 0.001), highlighting substantial variability in local practice patterns.

Comparatively, Banker et al. conducted a similar study in hospitalized patients and reported a 62% inappropriate repeat-testing rate for NT-proBNP within seven days. Importantly, 97% of repeat NT-proBNP measurements following an initially abnormal result did not provide clinically meaningful additional information [13]. Boerman et al. reported an inappropriate repeat-testing rate of 5.16% for NT-proBNP using a 30-day MRI threshold [9]. For comparison purposes, application of a 30-day MRI threshold to BNP in our cohort would have resulted in an inappropriateness rate of 42.3%. However, this discrepancy may partly reflect the educational and feedback interventions implemented before MRI enforcement in the study by Boerman et al., as well as differences in patient populations, biomarkers evaluated, and study design. Nevertheless, these findings illustrate the potential impact of targeted demand-management interventions and reinforce the multifactorial nature of laboratory test overuse.

Other biomarkers

Although the relationship was not strictly linear across clinical settings, a general trend was observed whereby the most frequently requested biomarkers tended to exhibit the highest rates of inappropriate repetition. One possible explanation is that these commonly requested biomarkers, often described as “routine tests,” are frequently included in predefined electronic order panels, which may reduce the need for clinicians to actively select the most appropriate biomarkers for a given clinical situation. Conversely, biomarkers with more specific clinical indications tend to be requested more selectively. This interpretation is further supported by the analysis of biomarkers grouped according to physiological categories. Indeed, the most selectively requested biomarker groups were also those with the highest compliance with MRI recommendations, namely, proteins, thyroid biomarkers, and serological biomarkers.

A representative example illustrating the differences between clinical settings is HbA1c. Due to its biological and clinical characteristics, the MRI threshold for HbA1c is identical across all three settings (three months). Nevertheless, the inappropriateness rate differed significantly between them (χ² test, p < 0.001; z-test for proportions, p < 0.001): 26.6% in inpatients, 18.7% in hospital outpatients, and 8.4% in primary care. Although there are clinical situations in which repeat testing before three months may help assess the trajectory of a decompensated diabetic patient, the magnitude of the rates observed, particularly in inpatients, appears difficult to explain based on current MRI recommendations and biological variation principles. In a hypothetical scenario applying a 30-day MRI threshold in our inpatient setting, the inappropriateness rate would decrease to 11.2%. For comparison, Bozyigit et al. reported inappropriateness rates of 9.9% in outpatients and 14.1% in inpatients using a three-month MRI threshold [10]. A review published by Lang reported an overall inappropriateness rate of 18.3% for HbA1c; however, these estimates were derived from pooled studies conducted across heterogeneous clinical settings and applying markedly different MRI thresholds (82-365 days) [15].

Economic impact

This study simulated the implementation of hard-stop MRI rules and projected a 14.2% reduction in total laboratory expenditure within our healthcare network, corresponding to approximately €119,281.93 per year. Notably, more than half of the estimated savings would be achieved by applying requisition restrictions to only two biomarkers. In the case of BNP, the high cost per test (€9.96) explains the large projected financial impact, accounting for 38.5% of the total cost reduction. In contrast, the estimated savings associated with complete blood count (15.7%) were a finding that was initially unexpected. Despite being a relatively inexpensive test (€0.57), it is the most frequently requested test in the laboratory. This high testing volume, combined with an overall repeat inappropriateness rate of 26.1%, explains the substantial projected savings.

Although the primary objective of this study was to evaluate inappropriate repeat testing outside MRI thresholds, inappropriate initial test ordering may also occur. A notable example is the prescription of procalcitonin in outpatient settings. This biomarker is valuable in the diagnosis of infection and sepsis and for guiding antibiotic therapy in hospitalized patients. However, it has no clear indication in stable outpatients without suspected infection. Notably, although the overall rate of inappropriate repeat testing for procalcitonin was only 0.9% (n = 248), a total of 834 measurements were performed in outpatient settings, corresponding to 3.1% of all procalcitonin requests. Considering that this is the second most expensive biomarker (€9.47 per test), and assuming that those outpatient requests were inappropriate, eliminating them would represent an additional potential annual saving of approximately €3,948.99. While seemingly modest, that would make procalcitonin the fifth biomarker with the greatest estimated savings. These findings suggest that addressing inappropriate initial test ordering may provide additional economic benefits beyond those achieved through MRI-based interventions alone.

More broadly, our findings highlight the value of systematic assessment of laboratory test utilization for optimizing laboratory demand management and improving the financial sustainability of healthcare institutions. Although laboratory testing accounts for less than 5% of total healthcare expenditure, it influences an estimated 60-70% of clinical decisions [3]. Consequently, the indirect economic and clinical impact of inappropriate testing likely exceeds the apparent savings from individual analyses. Given the inherent risk of false-positive results associated with any laboratory test, indiscriminate ordering increases the probability of spurious abnormalities, potentially triggering diagnostic cascades involving further testing, imaging, specialist consultations, or even iatrogenic harm. These downstream consequences represent a well-recognized yet frequently underestimated effect of laboratory overuse and support the view that adherence to MRI recommendations may contribute to both cost-efficiency and patient safety [2,3].

Laboratory demand management

From a practical perspective, several strategies could be considered to improve laboratory demand management within our institution. Beyond structural interventions such as hard-stop rules, additional targeted approaches may include real-time cost display at the point of test ordering; audit and feedback cycles with individualized clinician reports; integration of MRI-based alerts into electronic prescribing systems; and educational interventions focused on high-impact biomarkers identified in this study, such as BNP. Behavioral strategies, including subtle “nudging” mechanisms embedded in electronic health records, may further support more appropriate test utilization without restricting clinical autonomy.

Limitations

This study has several limitations that should be acknowledged. First, the analysis was based exclusively on laboratory information system data and did not include access to clinical records. Consequently, the clinical context of each test request could not be evaluated, and some repetitions classified as inappropriate according to MRI recommendations may have been clinically justified in specific circumstances. Second, MRI thresholds were derived from heterogeneous literature sources and expert consensus rather than universally standardized guidelines, which may introduce variability in the classification of appropriateness. Third, the economic analysis was limited to reagent costs and did not include indirect costs related to personnel time, laboratory infrastructure, or downstream healthcare utilization, meaning that the true economic impact of inappropriate testing may be underestimated. Finally, this study was conducted within a single healthcare network, and although it includes multiple hospitals and primary care centers, the findings may not be fully generalizable to other healthcare systems with different organizational structures or clinical practices.

Future research

Future research should focus on prospective evaluation of MRI-based interventions, including hard-stop and/or soft-stop policies, assessing not only reductions in test volume but also clinical outcomes, safety endpoints, and physician adherence. Additionally, studies integrating laboratory data with clinical records would allow more precise differentiation between appropriate and inappropriate test repetition.

## Conclusions

This study identified substantial non-compliance with MRI recommendations across multiple levels of care within a regional healthcare network, with the highest rates observed in the hospital inpatient setting. These findings suggest that inappropriate repeat laboratory testing remains a relevant systemic issue, particularly in acute-care environments, where diagnostic uncertainty, routine ordering practices, and institutional workflow may contribute to repeated testing with limited clinical value. By comparing primary care, hospital outpatient care, and inpatient/emergency settings within the same healthcare system, this study provides a comprehensive overview of how laboratory overuse varies according to clinical context. Importantly, the economic analysis indicates that a considerable proportion of potential cost savings may be achieved through targeted interventions focused on a limited number of high-impact biomarkers, particularly natriuretic peptides. Taken together, these findings support the implementation of MRI-based laboratory demand management strategies, including electronic decision-support tools such as hard-stop policies, combined with clinician engagement and locally adapted institutional measures. Although differences in healthcare organization and prescribing behavior may limit direct comparison with other settings, our results reinforce the value of proactive, context-sensitive approaches to improve test appropriateness, reduce unnecessary healthcare expenditure, and promote a safer and more sustainable model of laboratory medicine.
